# Will life find a way? Evolution of marine species under global change

**DOI:** 10.1111/eva.12418

**Published:** 2016-09-28

**Authors:** Piero Calosi, Pierre De Wit, Peter Thor, Sam Dupont

**Affiliations:** ^1^ Département de Biologie Chimie et GéographieUniversitè du Québec à RimouskiRimouskiQCCanada; ^2^ Department of Marine SciencesUniversity of GothenburgStrömstadSweden; ^3^ Norwegian Polar InstituteFram CentreTromsøNorway; ^4^ Department of Biological and Environmental SciencesUniversity of GothenburgFiskebäckskilSweden

**Keywords:** marine evolution, ocean warming, ocean acidification, salinity, phenotypic plasticity, local adaptation, rapid adaptation, epigenetics, evolutionary modeling, transgenerational responses, DNA methylation, common garden experiment

## Abstract

Projections of marine biodiversity and implementation of effective actions for its maintenance in the face of current rapid global environmental change are constrained by our limited understanding of species’ adaptive responses, including transgenerational plasticity, epigenetics and natural selection. This special issue presents 13 novel studies, which employ experimental and modelling approaches to (i) investigate plastic and evolutionary responses of marine species to major global change drivers; (ii) ask relevant broad eco‐evolutionary questions, implementing multiple species and populations studies; (iii) show the advantages of using advanced experimental designs and tools; (iv) construct novel model organisms for marine evolution; (v) help identifying future challenges for the field; and (vi) highlight the importance of incorporating existing evolutionary theory into management solutions for the marine realm. What emerges is that at least some populations of marine species have the ability to adapt to future global change conditions. However, marine organisms’ capacity for adaptation appears finite, due to evolutionary trade‐offs and possible rapid losses in genetic diversity. This further corroborates the idea that acquiring an evolutionary perspective on how marine life will respond to the selective pressure of future global changes will guide us in better identifying which conservation efforts will be most needed and most effective.

<<It is difficult to believe in the dreadful but quiet war lurking just below the serene facade of nature>> (Charles Darwin 1859)

The chemical and physical evidence for ongoing anthropogenic global change is now so prevalent that the conclusion that our climate is drastically changing is considered indisputable (IPCC 2013). On the other hand, and despite the tremendous effort by the Intergovernmental Panel on Climate Change (IPCC 2013) to synthesize our present understanding of the biological implications of global change, biological evidence corroborating the existence of ubiquitous mechanisms governing species’ responses to future environmental challenges is somewhat lagging behind (Melzner et al. 2009; Dupont and Pörtner 2013; Kroeker et al. 2013; Wittmann and Pörtner 2013; Storch et al. 2014). This discrepancy has so far prevented us from producing more conclusive projections on the fate of living systems under global change. What appears to be certain is that we are on the brink of a global biodiversity crisis (Barnosky et al. 2011). It is thus unlikely that any extant species and ecosystem will be able to survive the ongoing planetary environmental changes without actually changing. In fact, whilst migration can temporarily help prevent a species’ global extinction, ultimately it is only through evolutionary adaptation that populations and species can be rescued from local and global extinction (Gonzalez et al. 2013). Nonetheless, phenotypic plasticity may buy additional time for adaptation to occur (Godbold and Calosi 2013; Munday et al. 2013; Reusch 2014; Sunday et al. 2014) and also provide a mechanism for adaptation to occur rapidly (Pigliucci et al. 2006; Ghalambor et al. 2015). Finally, extant levels of adaptation to local conditions may mediate populations’ sensitivity to future global change drivers (e.g. Lardies et al. 2014; Wood et al. 2016). For these reasons, the investigation of populations’ and species’ ability to mount plastic and adaptive responses to prevalent environmental changes is an absolute priority (e.g. Pespeni et al. 2013), if we are to identify which populations, species and assemblages will survive global change, and which are more likely to go extinct (Hall‐Spencer et al. 2008; Calosi et al. 2013; Lucey et al. 2015). As current efforts have to a large extent focused on individual species’ abilities to cope with short‐term changes through plastic responses, in order to make critical predictions of long‐term responses, it is essential to gain an understanding of the mechanisms behind the complex interactions between plasticity, evolution and nongenetic inheritance (epigenetics).

The study of evolution is one of the central themes of modern biology (Darwin 1859; Dobzanshky 1937, Huxley 1942; Dobzhansky 1937; Margulis 1999; Noble 2015). Species’ capacity to mount evolutionary responses to fluctuations and changes in the environment has been investigated for decades both *via* comparative (see Somero and Hochachka 2002; Stillman and Paganini 2015) and correlative methods (see Colin and Dam 2002; Gaston et al. 2009; Bozinovic et al. 2011; Dam 2013). More recently, in order to overcome some of the limitations of these former methods, the implementation of experimental evolutionary methods has been favoured (e.g. Bennett et al. 1992; Garland and Rose 2009; Kellermann et al. 2009). Nonetheless, in the field of marine global change biology, the investigation of the capacity of biological systems to adapt to the ongoing rapid environmental change has been largely overlooked, at least until very recently (Godbold and Calosi 2013; Munday et al. 2013; Reusch 2014; Sunday et al. 2014). This situation may result from the historical lack of true marine model systems, particularly for multicellular organisms when compared to terrestrial systems (e.g. *Drosophila*,* Arabidopsis*). In part, this has been a consequence of the difficulty of working with long‐lived species, in a poorly understood environment, as well as having to deal with maintaining desired environmental conditions in laboratory sea water. Nonetheless, as marine systems, just like other biological systems, are intrinsically plastic (Ghalambor et al. 2007) and have the ability to evolve (Darwin 1859), in some cases rapidly (e.g. Ghalambor et al. 2015; Thor and Dupont 2015), these features can no longer be ignored when trying to project the responses of marine populations, species and assemblages to rapid changes in multiple environmental drivers.

There is no doubt that the IPCC (2013) has generated an in‐depth synthesis of the patterns through which marine species and ecosystems presently respond to the ongoing global change and may do so in the future (Pörtner et al. 2014). However, if we are to critically improve current predictions of the fate of global biodiversity under the current environmental change, advances in understanding of the drivers and mechanisms behind marine evolution are required. In this sense, the investigation of trans‐generational plastic and evolutionary responses of fitness‐related traits under global change scenarios, and the identification of the underpinning physiological genetic and nongenetic mechanisms, is central to advance our current understanding of how marine organisms will be able to cope with future environmental challenges. Using an evolutionary approach will help us avoid potential overestimations or underestimations of the biological implications of global change (Dam 2013).

Consequently, this special issue aims to collect novel, cutting‐edge studies, which represent a further proof for the idea that the investigation of evolution within the context of marine global change is imperative, and can help guiding environmental management and conservation solutions under the ongoing rapid global change.

The specific objectives of this special issue are to (i) investigate plastic and evolutionary responses of ecologically important marine species to some of the major global change drivers (e.g. ocean warming, ocean acidification, salinity changes), both as single drivers but also combined (simultaneous and sequential); (ii) move towards asking relevant broad eco‐evolutionary questions, implementing well‐designed multiple species and populations studies; (iii) show the advantages of using advanced experimental designs and appropriate tools (from high‐throughput DNA sequencing and novel methods for studying methylation patterns, to mathematical modelling); (iv) move beyond current limitations by constructing novel model organisms for evolution in the marine realm; (v) help identify some of the future challenges for the field of marine global change biology; and finally (vi) highlight the importance of incorporating existing evolutionary theory into management solutions for the marine realm.

This special issue consists of thirteen original manuscripts, focusing on unicellular organisms, macroalgae, invertebrates and vertebrates as study models. Most importantly, these works cover a broad range of approaches and topics relevant to the development of marine global change research. These include (i) the investigation of the significance of local adaptation in defining populations’ responses; (ii) the importance of trans‐ and multigenerational responses to mediate species’ plastic responses; (iii) the possibility for rapid evolution to occur; and (iv) the relevance of epigenetic mechanisms, as well as evolutionary trade‐offs, in mediating species’ responses. From these studies, a number of relevant messages and lessons have emerged and are briefly summarized below.

## Local adaptation

A species’ level of local adaptation, here defined as the process of evolution of a given population in response to the prevalent local environmental regimes (Williams 1966) in the face of gene flow from nearby populations, will be critical to define populations’ responses to future environmental conditions, by either providing a buffer for future negative impacts, or increasing sensitivity levels (e.g. Sanford and Kelly 2011; Calosi et al. 2013; Dam 2013; Pespeni et al. 2013; Savolainen et al. 2013). Within this special issue, Padilla‐Gamiño et al. (2016) have used multiple life stages of different species of coralline algae to test the hypothesis that populations living in habitats characterized by higher variability and elevated levels of seawater *p*CO_2_ will be less affected by future ocean acidification, when compared to populations from habitats characterized by more stable and low levels of seawater *p*CO_2_. They were able to show that spores are less sensitive to elevated *p*CO_2_ than adults, and reported more marked impacts in populations found in habitats characterized by lower variability and lower levels of seawater *p*CO_2_. These findings have important implications for the conservation of these important ecosystem engineers in the future ocean.

On the other hand, Lucey et al. (2016) carried out a reciprocal transplant on individuals of the sessile calcifying polychaete *Simplaria* sp. from a population inhabiting a naturally elevated *p*CO_2_ volcanic vent area and a population from a nearby control area exposed to unaltered water chemistry conditions. Their results indicate that in this taxon neither local adaptation nor phenotypic plasticity may suffice to buffer the negative impacts of future ocean acidification. In more detail, Lucey et al. (2016) showed that regardless of their original environmental conditions, both populations showed low fitness levels, increased tube growth rates and similar plastic responses when exposed to elevated *p*CO_2_ conditions, suggesting that local adaptation to a low pH environment had not occurred and that long‐term exposure had not caused any substantial phenotypic changes.

Results from these two studies suggest that local adaptation to future conditions may not be a ubiquitous process in the marine environment. Large variability in evolutionary and plastic responses may exist, most likely resulting from differences in life‐history strategies, population size, fecundity and gene flow. Understanding the relative contributions of these parameters to local adaptive capability will enable us to widen our knowledge on the importance of the process of adaptation to counter environmental change, and ultimately use it to promote the conservation of marine biodiversity. Indeed, the investigation of local adaptation must become a conservation and resource management priority (Lucey et al. 2016). Finally, Padilla‐Gamiño et al. (2016) and Lucey et al. (2016) both show the value of comparing populations living under differing environmental regimes as an approach to study marine organisms potential for evolution under global change.

## Trans‐generational and multigenerational studies, and evidence for rapid selection

Trans‐generational effects, defined as changes in offspring phenotype due to stress exposure of the parental generation, have the potential to buffer species against environmental changes (Sunday et al. 2014). In this special issue, Donelson et al. (2016) use a model coral reef fish (*Acanthochromis polyacanthus*) to investigate the impact of different heat exposure of parents on the next generation's reproductive output ability and the quality of offspring produced. Interestingly, they found that a gradual warming over two generations resulted in greater plasticity of the reproductive traits investigated, when compared to fish that experienced the same increase within one generation. Similarly, evidence for positive effect of trans‐generational exposure in helping restabilizing reproductive output levels following a rapid change in *p*CO_2_ is also provided by Rodriguez‐Romero et al. (2015), using a laboratory strain of an emerging marine polychaete model (*Ophryotrocha labronica*). These studies (Donelson et al. 2016; Lucey et al. 2016) suggest that trans‐generational plasticity can induce full restoration of fitness‐related traits, which may not be observed with developmental plasticity alone. Furthermore, Rodriguez‐Romero et al. (2015) also conducted a mutual transplant experiment, following seven generations of exposure to differing *p*CO_2_ conditions, providing evidence for the possible occurrence of rapid adaptation in a marine organism to rapid environmental change. Rodriguez‐Romero et al. (2015) show the importance of conducting multigenerational experiments in order to provide more realistic estimates for marine metazoans’ responses to future environmental changes. However, they also highlight the limitations of interpreting the evolutionary significance of the outcome of transgenerational and multigenerational experiments, without the use of physiological and genetic tools, often not available for nonmodel organisms.

A number of studies in this special issue integrate novel physiological and genetic tools in the investigation of marine organisms’ responses to global change drivers. For example, Shama et al. (2016) investigated differences in mitochondrial respiratory capacity and gene expression across three generations in marine sticklebacks (*Gasterosteus aculeatus*) exposed to heat stress, either in an acute fashion or throughout development, allowing for some acclimation to occur. They used an advanced cross‐breeding experimental design and demonstrated that the mechanisms underlying trans‐generational effects persist across multiple generations, leading to phenotypes for mitochondrial respiratory capacity and gene expression that depend on both the type of acclimation and the environmental mismatch between generations. In addition, De Wit et al. (2015) further corroborated the evolutionary significance of the mitochondrial function in underpinning species’ transgenerational responses to global changes. In order to do this, they exposed specimens of the copepod *Pseudocalanus acuspes* to different *p*CO_2_ conditions over two successive generations, followed by a reciprocal transplant experiment (Thor and Dupont 2015). After this, they used a physiological hypothesis‐testing strategy to mine both gene expression and nucleotide sequence data showing that exposure to elevated *p*CO_2_ appears to impose selection in copepods on both mitochondrial and ribosomal function, and that these changes might be related to changes in RNA transcription activity. The important consequence of this work is that De Wit et al. (2015) show that evolution of fitness‐related traits can occur rapidly in marine metazoans exposed to future global change scenarios, especially in species with high standing genetic variation and large population sizes. This gives some hope that selection acting on exiting phenotypic and genetic diversity can promote the rescue of some marine metazoans within the context of future global change conditions (Munday et al. 2013; Reusch 2014; Sunday et al. 2014).

Genetic diversity could rapidly diminish in the face of rapid environmental changes, as shown by Lloyd et al. (2016) in the larvae of the purple sea urchin (*Strongylocentrotus purpuratus*) exposed to elevated *p*CO_2_ conditions. Lloyd et al. (2016) showed a greater loss of nucleotide diversity under elevated *p*CO_2_ conditions than in control settings, and the authors suggest that in wild populations, loss of genetic diversity could limit their capacity for further adaptation to future ocean acidification, or other drivers, in future generations. The authors concluded that whilst some natural populations may currently possess sufficient standing genetic variation to face future global changes, this latent ability of populations to deal with future environmental challenges may be rapidly dissipated by the ongoing environmental change.

Chakravarti et al. (2016), using an emerging marine polychaete model (*Ophryotrocha labronica*), exposed individuals to projected ocean warming and acidification conditions over successive generations, and showed that trans‐generational exposure in the laboratory can improve offspring fitness under single driver exposure, but not across all traits measured, potentially due to genetic or physiological constraints or trade‐offs. In addition, Chakravarti et al. (2016) found no significant effect of exposure to combined global change drivers. As a consequence, the utilisation of human‐assisted acclimation may require an in depth reflection before local and global proactive conservation plans are put into motion (Van Oppen et al. 2015).

The existence of trade‐offs between tolerance traits to different stressors can limit both species’ plastic and evolutionary responses (e.g. Hoffmann and Sgrò 2011; Dam 2013). In order to test this idea, Kelly et al. (2016) hybridized (here intended specifically as crossings) different populations of the intertidal copepod Tigriopus californicus, differing for both heat and salinity tolerance, and undertook a multigenerational selection experiment for tolerance to heat, hypo‐osmotic and hyperosmotic conditions. They found that (i) heat‐selected lines were more heat tolerant but showed lower fecundity, (ii) hyperosmotic‐selected lines showed a reduction in tolerance to heat and (iii) lines selected for both heat and hypo‐osmotic stress combined showed a reduced tolerance to heat, thus indicating, together with transcriptomic evidence, that energy trade‐offs exists for these two tolerance traits.

Finally, in an impressively long‐lasting selection experiment, Listmann et al. (2016) investigated changes in thermal reaction norms in the model calcifying coccolithophore *Emiliania huxleyi* in response to 2.5 years of experimental selection to two temperatures (1200 asexual generations). The different thermal selection regimes led to a marked divergence of thermal reaction norms for optimal growth and maximum persistence temperature to a range of temperatures and *p*CO_2_. Altogether, Listmann et al. (2016) showed that thermal reaction norms in phytoplankton may evolve at a faster pace than that of predicted ocean warming, bringing some hope for the future of a key element of marine ecosystems.

## Epigenetics responses

Among the mechanisms underlying both plastic and evolutionary processes, especially trans‐generational effects, epigenetic mechanisms (e.g., DNA methylation or histone modification) have to date been understudied within the context of marine organisms’ responses to global change (Bonduriaski et al. 2012). This may have been primarily caused by the lack of well‐developed model organisms and tools for the marine realm, as well as the relatively recent discovery of the importance of these mechanisms. However, many current initiatives address this issue, with new technological advances making it possible to study epigenetic patterns even in less‐than‐fully developed model systems. Taking advantage of these recent advances, Putnam et al. (2016) tested whether scleractinian corals of the environmentally sensitive species *Pocillopora damicornis* and more environmentally robust species *Montipora capitata* exhibited differences in their phenotypic response that were associated with changes in DNA methylation levels following exposure to elevated *p*CO_2_. Putnam et al. (2016) showed that the more sensitive species exhibited a reduced calcification rate under elevated *p*CO_2_, which was not seen in the more tolerant species. In addition, the sensitive species exhibited larger changes both in its metabolomic profile and DNA methylation pattern, when compared to the most robust species. This novel study highlights the relevance of investigating environmentally induced changes in DNA methylation, as mechanisms mediating the responses to major global change drivers of important ecosystem engineers, such as are corals, whilst asking relevant broad eco‐evolutionary questions. This line of investigation could provide us with a tool to generate heritable plasticity, in support of future conservation actions, and to promote assisted evolution in marine organisms (Van Oppen et al. 2015). It will be critical to focus future work on the relationship between methylation patterns, gene expression and evolution in the generation of the observed phenotypic trans‐generational effects that might provide a rescue mechanism for species facing global change.

## The modelling approach

Experimental and field observational approaches have so far led the way in building our understanding of how future marine biotas will be shaped by ongoing environmental changes (Godbold and Calosi 2013; Munday et al. 2013; Reusch 2014; Sunday et al. 2014). Mathematical models may provide conceptual frameworks within which such experimental data can be placed in context. Further, models can be used as tools in order to design well‐informed and well‐designed experiments to produce much needed proof of concepts for key aspects of biological systems responses to the global change.

Using an individual‐based model, Collins (2016) investigated the evolution of cell division rates in asexual populations of unicellular microbes maintained under chronic environmental nutrient enrichment over hundreds of generations. She found that after many generations, initially elevated growth rates appear to become limited by increases in cellular damage. This in turn causes the growth rates to decline to the ancestral state, which Collins (2016) calls the ‘Prodigal Son dynamics,’ in the absence of further evolution for increased tolerance to damages or decreasing in repair cost or decreasing in rate of damages. An implication from this work is that a continuous increase in growth rate, usually taken as a sign of increased fitness, might actually be detrimental to a population in the long run and that intermediate rates are more sustainable and are positively selected for. This theoretical approach is relevant to inform our understanding of how environmental enrichment can increase or control cell division rate in a sustainable fashion, these processes being central to important applications such as biofuel reactors and controlling biofouling, respectively.

Finally, Marshall et al. (2016) used a heuristic model to explore how traits associated with complex life histories, often found in marine organisms, can alter a population's capacity to cope with environmental change. Marshall et al. (2016) found that an increase in life‐history complexity decreases the potential for evolution of a species during environmental change. The authors go further, suggesting that levels of genetic correlations in stress tolerance between different life stages, genetic variance levels characterizing each life stage and the relative plasticity level found among different stages, all interact to determine the environmental change threshold any given species can tolerate before extinction occurs. Marshall et al. (2016) concluded based on their model that marine organisms possessing more complex life cycles are particularly sensitive to future global change drivers, but also warn us that for most species we still have to acquire experimental evidence for key traits.

A broader implementation of relatively simple models such as those developed by Collins (2016) and Marshall et al. (2016) could, if well employed and further parameterized with empirical data, rapidly improve our understanding on both specific trait responses and biodiversity responses to the global change.

## Conclusion

This special issue collects a number of novel cutting‐edge studies showcasing advanced experimental designs, approaches and tools to be used in the investigation of key aspects of marine organisms’ evolutionary responses to ongoing and future rapid global changes. This new knowledge further demonstrates that ‘Life may find a way’, that is at least some populations of some marine species have the ability to adapt to future global change conditions, and illustrates, through transgenerational and epigenetic studies, some of the evolutionary pathways and mechanisms of adaptation that may occur over the next decades. At the same time, we have seen that the potential and capacity of marine organisms for adaptation are finite, due to the presence of evolutionary trade‐offs among different traits, particularly when exposed to multiple global change drivers, and the possibility that extant genetic diversity, which enable populations to adapt to changing environments, is quickly reduced with ongoing environmental changes. Consequently, extinction caused by global change in some populations and species in the marine realm, particularly for metazoans, can be expected. This critical understanding of how marine organisms will change under the selective pressure of future global change drivers should be harnessed to help us better predict population‐, community‐ and ecosystem‐level responses. This is particularly relevant when considering the discrepancy between our current understanding of the rate of change for environmental parameters versus the rate of change (through plasticity and adaptation) of biological systems, within the context of the ongoing global change (Dam 2013; Pörtner et al. 2014). Further, studies of species’ local adaption, their capacity for trans‐ and multigenerational plasticity and rapid evolution, and the existence of epigenetic responses mediating species plasticity need to be increasingly incorporated into future models of evolution under global change. Such studies will provide powerful tools in our efforts to promote marine conservation and provide increasingly reliable projections on changes in marine biodiversity in the face of global change. At the same time, field observations aiming at detecting ongoing biological changes will be critical to assess whether plasticity and adaptation responses observed under laboratory conditions are actually observed in nature (Garland and Rose 2009), and are occurring at a rate which is fast enough to prevent local and global extinction. This integration will further support our ability to produce reliable projections on changes occurring from the species to the ecosystem level. Current evidence appears to suggest that plasticity and adaptation may not be fully effective in promoting evolutionary rescue (e.g. Pörtner et al. 2014), as past mass extinctions may also suggest, particularly considering the rapidity of the ongoing environmental change (Barnosky et al. 2011). Nonetheless, relevant evolutionary information will guide us in identifying which conservation efforts may be the most needed to prevent populations and species extinction and the most effective, i.e. epigenetic manipulation, laboratory transgenerational exposure, artificial selection (Van Oppen et al. 2015). Furthermore, this approach will help us identify what rate and magnitude of environmental change we can afford for life to be able to eventually adapt; in turn, which are the thresholds for the rate of environmental change beyond which evolution will not be effective in rescuing marine organisms? We hope that future efforts (including those by the IPCC) will increasingly incorporate our current, and rapidly increasing, knowledge on marine biological systems’ evolutionary responses to rapid environmental changes.

## Author contributions

All authors contributed to the rational for this work. PC, SD and PDW wrote the first draft of this manuscript, and all authors contributed to the final write‐up.

## Funding

PC was funded by the NERC UK Ocean Acidification Research Programme (NE/H017127/1), and he is supported by an NSERC Discovery Grant and a FRQNT New University Researchers Start‐Up Program. PDW was supported by a postdoctoral grant from the Marcus and Amalia Wallenberg Foundation, as well as BONUS, funded jointly by the EU and the Swedish FORMAS. PT was funded through the ‘Ocean Acidification and Ecosystem Effects in Northern Waters Flagship’ from the High North Research Centre for Climate and the Environment (Fram Centre). SD was financially supported by the Linnaeus Centre for Marine Evolutionary Biology at the University of Gothenburg (http://www.cemeb.science.gu.se) and a Linnaeus grant from the Swedish Research Councils VR and Formas.
